# Lipoxin-Induced Phenotypic Changes in CD115^+^LY6C^hi^ Monocytes TAM Precursors Inhibits Tumor Development

**DOI:** 10.3389/fonc.2019.00540

**Published:** 2019-06-19

**Authors:** Natália Mesquita de-Brito, Hayandra Cunha da-Costa, Rafael Loureiro Simões, Christina Barja-Fidalgo

**Affiliations:** Laboratory of Cellular and Molecular Pharmacology, Department of Cell Biology, Universidade do Estado do Rio de Janeiro, Rio de Janeiro, Brazil

**Keywords:** cancer, tumor-bearing mice, tumor-associated macrophage, ATL-1, monocyte

## Abstract

During tumor development, the spleen acts as an extra-medullar reservoir of LY6C^hi^ inflammatory monocytes, which can migrate toward tumor to differentiate into tumor-associated macrophage (TAMs), renewing the TAM population. In the tumor microenvironment, pro-inflammatory macrophages (M1) acquire anti-inflammatory and pro-tumor (M2) characteristics favoring tumor development. We previously demonstrated that lipoxins, a family of pro-resolving lipid mediators, restored *in vitro* the cytotoxic M1-like properties of TAMs.

**Objective:** In this study, we have investigated *in vivo* the cellular mechanisms underlying the anti-tumor property of lipoxins.

**Methods:** Fourteen days after inducing B16-F10 melanoma tumors, mice received one single dose of ATL-1 (1 μg/i.v.), a lipoxin A4 analog. After further 7 days, blood and bone-marrow were collected, tumors and spleens were removed, and TAMs and blood monocytes were isolated.

**Results:** While the population of LY6C^hi^ monocytes was increased in non-treated tumor-bearing mice, the treatment with ATL-1 diminished the population of LY6C^hi^ monocytes in spleen, blood and bone marrow, decreasing macrophage infiltration into the tumor and reducing the M2 markers expression on TAMs. Importantly, those effects were accompanied by an impairment of tumor growth and improved survival of tumor-bearing mice. The data evidence the anti-tumor mechanism of ATL-1, by decreasing the availability of TAM-precursor monocytes and changing TAMs profile *in vivo*, impairing tumor progression. ATL-1 may become a new tool in cancer control.

## Introduction

Tumor progression shares basic mechanisms with inflammatory process (1), such as the presence of inflammatory cells and mediators released by both tumor cells and infiltrated leukocytes (2). When activated, monocyte-derived macrophages may assume different phenotypes, depending on the type of stimulus. In general, the classical M1 activation is characterized by the efficient production of reactive oxygen and nitrogen species, as well as the production of proinflammatory cytokines (IL-1β, TNF-α, and IL-6), effectively participating as inducers and effectors of the response Th1. Conversely, M2 or alternatively activated macrophages produce higher amounts of anti-inflammatory cytokines and participate in type 2 (Th2) reactions, inducing remodeling and tissue repair (3, 4). When activated by molecules present in the tumor environment, these macrophages present a functional profile type M2, also called TAM (tumor-associated macrophage) (4–6).

TAMs are among the most versatile tumor-infiltrating inflammatory cells and may represent around 50% of the non-cancer cells of the tumor mass. They participate in all hallmarks of cancer by generating numerous growth factors, cytokines and extracellular matrix (ECM)-remodeling molecules, that regulate tumor growth, migration and angiogenesis (7–9). Evidence have shown that an increased number of M2-like TAMs correlates with poor prognosis in different cancers types both in murine models and human patients (10, 11).

The macrophage population found in the inflammatory sites are usually derived from monocytes coming from distinct cellular niches, prevailing the bone marrow and spleen (12–15). The spleen constitutes a unique extramedullary reservoir of myeloid cells, mainly monocytes (16), and in cancer, the extramedullary hematopoiesis in the spleen may support the continuous supply of monocytes to the tumor (17, 18). The maturation of different monocyte subsets can result in the induction of differential macrophage profile. In mice, monocytes (CD11b^+^CD115^+^) are grouped in subsets according to the expression levels of Ly6C on cell surface. The Ly6C^hi^ monocytes present pro-inflammatory functions, while the Ly6C^low^ cells, are responsible for patrolling and tissue repair. Additionally, these subsets can be further classified including others surface markers and chemokine receptors. Ly6C^hi^ subsets are usually CCR2^hi^CX3CR1^low^, while Ly6C^low^ monocytes are CCR2^low^CX3CR1^hi^ (19). During chronic inflammation, the spleen can continuously contribute with Ly6C^hi^ monocytes and Ly6G^hi^ granulocytic cells (20). It has been reported that Ly6C^hi^ “inflammatory” monocytes were the precursors of all the distinct TAM subsets in a mouse mammary tumor model (21). In this context, modulation of these TAMs precursors cells can constitute a powerful tool to control tumor progression.

Lipoxins (LX) are specialized pro-resolving lipid mediators that are down-regulated in certain tumors. They display potent anti-inflammatory actions, modulating selective effects on monocytes and macrophages, *in vitro* and *in vivo*, acting as a potent chemoattractant and promoting phagocytic clearance of apoptotic cells (22–25). Recently, our group demonstrated that ATL-1(15-epi-16-(para-fluoro) phenoxy-LXA_4_), a more stable analog of 15-epi-lipoxinA_4_, down-modulates the pro-tumor activity of TAMs *in vitro*, inducing a shift from the M2-like profile to a M1-like cytotoxic profile, leading to tumor cell death *in vitro* (26).

Despite the evident antitumoral role of lipoxin on TAM phenotype and functional profile, the effects exerted *in vivo* by this lipid mediator on TAMs precursors at the different sites of cell maturation are unknown. In the present study, we show that one single administration of a lipoxin analog decreases CD115^+^LY6C^hi^ monocyte population (TAM precursors) in the bone marrow, spleen and blood of animals with tumor. Additionally, ATL-1 treatment induces a *in vivo* switch in TAMs profile, from M2- to M1-like cells, improving the survival of tumor-bearing mice and decreasing tumor growth.

## Materials and Methods

### Melanoma Cell Culture

B16F10 murine melanoma cells, obtained from American Type Culture Collection (ATCC), were maintained in RPMI enriched with 10% fetal bovine serum, 3.7 g/L sodium bicarbonate, 5.2 g/L HEPES, 0.5 U/mL penicillin, and 0.5 mg/mL streptomycin at 37°C in a 5% CO_2_ humidified atmosphere. The number of cells present in cell suspension was determined by counting in Neubauer's chamber. Cell viability was determined by Trypan Blue (Gibco Invitrogen Corporation) exclusion.

### Murine Melanoma Model

All experiments on animals were conducted according to the principles of the NIH Guide for the Care and Use of Laboratory Animals and were approved by the Committee for the Ethics of Animal Experimentation of the Universidade do Estado do Rio de Janeiro (UERJ, Permit number: CEA/074/2012). Mice, males C57BL/6 at 6–7 weeks of age, weighing 25–30 g, were provided by the Animal House of the Department of Pharmacology and Psychobiology of UERJ. The animals were kept in cages (5 mice per cage) with free access to food and fresh water in a temperature-controlled room (22–24°C) on a 12 h light/dark cycle. For tumor induction, B16F10 melanoma cells (2 × 10^5^ cells/mouse suspended in 100 μL of saline) were inoculated subcutaneously (s.c.) into the right flank. After 14 days, when the tumors were visibly similar, were injected intravenously with ATL-1 (1 μg/mouse) or with ethanol (Vehicle). For experimental analysis, animals were sacrificed on day 21 after melanoma induction.

### Isolation of TAMs and Monocytes

On the 21st day after melanoma cell injection, animals were anesthetized (Ketamine 20 mg/kg + Xylazine 50 mg/kg) and blood was withdrawn by cardiac puncture. After that, animals were euthanized, and the tumors, spleen, and bone marrow were surgically removed. These removed tissues were immediately incubated for 30 min at 37°C with collagenase type IA (0.07%, Sigma®). Digested tissues were macerated in a cell strainer (100 μm pore, BD Falcon®), and the collected macerated was centrifuged at 1,200 rpm at 4°C with acc 8 for washing and suspended in 5 mL PBS. For mononuclear cells separation, the cell suspension was added to 5 ml of Ficoll-Hypaque® and centrifuged (400 × g/4°C/acc 5) for 40 min. The mononuclear cell layer was collected and washed 2 times (1,200 rpm/4°C/acc 8). Subsequently, the pellet was suspended, and the total number of mononuclear cells was quantified by light microscopy.

### Flow Cytometry

For surface marker analysis, TAMs obtained from tumor and monocytes from the spleen, blood and bone marrow were suspended in flow cytometry buffer (1 × 10^6^ cells/mL) and labeled with anti-mouse antibodies F4/80-APC, anti-CD206-PE, anti-CD115-PE, anti-LY6C-FITC. The Supplementary Figure 1 shows the gating strategies used to select the population of interest: monocytes (Supplementary Figure 1A) and tumor macrophages (Supplementary Figure 1B). Isotypes were used as control and the experiments were performed using the same number of cells (events) in all experimental groups. Fluorescence-activated cell sorting (FACS) analysis was conducted with an ACCURI C6 flow cytometer with CFLOW software (Becton Dickinson, Heidelberg, Germany).

### mRNA Expression (RT-qPCR)

Total RNA of TAMs extracted from tumors was isolated using the RNeasy Mini Kit (Qiagen®), according to the protocol provided by the manufacturer. RNA samples were stored at −80°C until use. The quality and quantity of RNA were analyzed by absorbance in NanoVue Plus (GE Healthcare®) and subsequently treated with DNAse. After the treatment, RNA samples were retro-transcribed into complementary DNA using the High Capacity cDNA Reverse Transcription kit (Applied Biosystems®). The complementary DNA samples were stored at −20°C until use. The cDNA was amplified using primers: Arginase-1, NOS2 and Actin (Qiagen®). The expression level of each gene analyzed was normalized by Actin.

The qPCR was performed on Gene Q Rotor and the amplicons were quantified by the SYBR-green fluorescence system (Qiagen®). The standard condition of the PCR reaction was: 95°C for 5 min, followed by 40 cycles at 95°C (5 s) and 60°C (10 s), followed by a standard denaturation curve.

### Cell Extracts

Macrophages isolated from the tumor-bearing mice treated or not with ATL-1 were resuspended in RIPA lysis buffer added with protease inhibitors. The protein content of the total extract was determined by the BCA method (Thermo Scientific®). Subsequently, 20% of sample buffer five times concentrated was added to each sample, and then boiled for 5 min for denaturation.

### Immunoblotting

The volume of cell extracts corresponding to 20 μg of protein was subjected to SDS-PAGE electrophoresis. Proteins present in the gel were transferred to a PVDF membrane (GE Healthcare®). Membranes were blocked with 5% BSA solution in Tris-buffered saline (TBS) containing 0.1% Tween-20 (T-TBS), and incubated overnight at 4°C with anti-arginase-1 or anti-actin (1: 1,000). After 3 5-min washes with T-TBS, the membranes were incubated with peroxidase conjugated secondary antibodies (1: 10,000) for 1.5 h and incubated with chemiluminescent solution (ECL Prime Western Blotting Detection Reagent Kit-GE Healthcare). The immunoreactive bands were visualized in the ChemiDoc MP Imaging System (Bio-Rad®). Images were analyzed with ImageJ software (NIH/USA).

### Statistical Analysis

Results are expressed as mean ± S.E.M for independent observations, where *n* represents the number of individual mice in each treatment group. Significance was obtained through the ANOVA test, followed by the Bonferroni test, or by the *t*-student test, where *p* < 0.05 was considered statistically significant.

## Results

### Tumor-Bearing Mice Present an Increase in Circulating Population of CD115^+^LY6C^hi^ Monocytes

**Figure 1:**
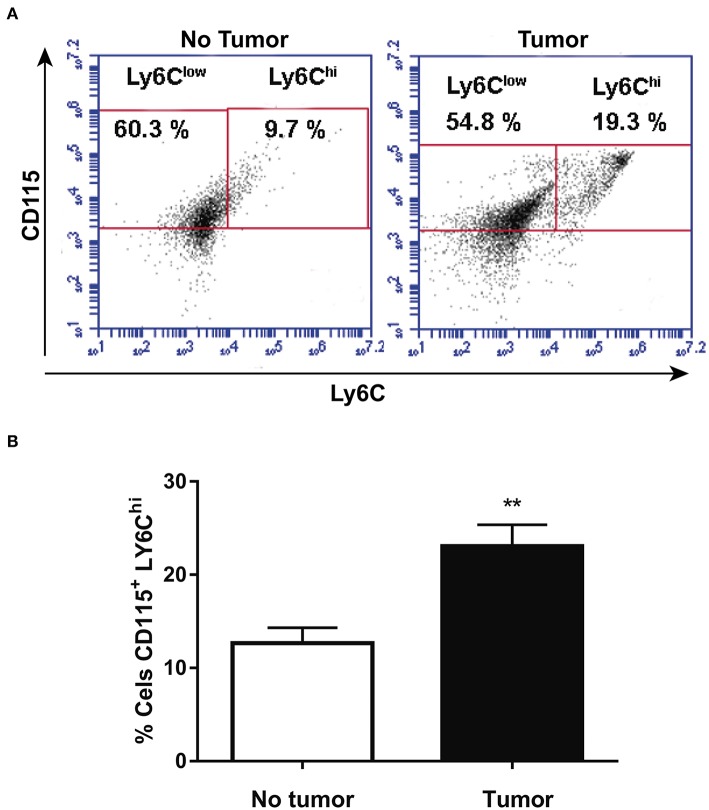
Tumor-bearing mice present increased circulating population of CD115^+^LY6C^hi^ monocytes. After 14 days, animals with or without tumor, were anesthetized and blood was withdrawn by cardiac puncture. Thereafter, monocytes were isolated from the blood and analyzed by flow cytometry. **(A)** Representative dot plot used to quantify the percentage of CD115^+^/LY6C^hi^ population within the selected gate. **(B)** CD115^+^/LY6C^hi^ population (*n* = 6). Data are expressed as mean ± SEM. **p < 0.01 by unpaired *t*-test.

CD115 and Ly6C expression was analyzed by flow cytometry in blood monocytes isolated from health animals or at 14th day after melanoma cells injection. Figures 1A,B show that tumor-bearing mice present higher population of CD115^+^LY6C^hi^ blood monocyte than health animals. The data corroborates other studies (21) and reinforce the suggestion that inflammatory LY6C^hi^ monocytes may be TAM precursors in circulation.

#### ATL-1 Treatment Decreases CD115^+^ LY6C^hi^ Cell Population in Bone Marrow, Spleen, and Blood of Tumor-Bearing Mice

**Figure 2:**
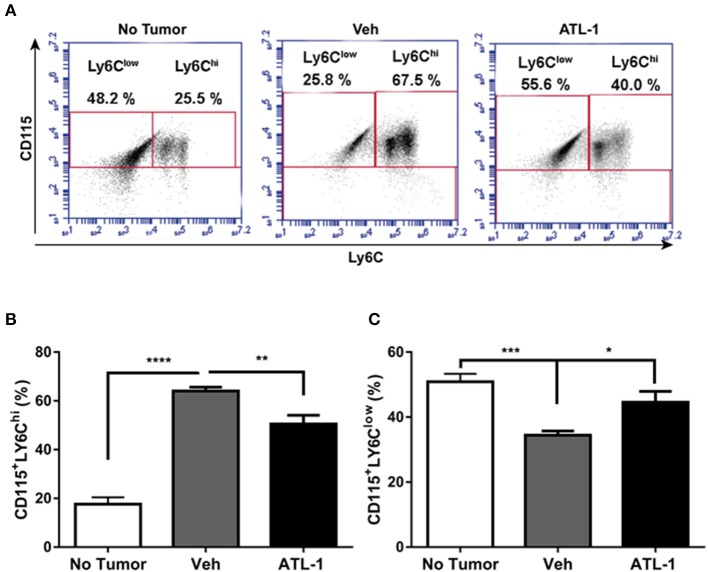
ATL-1 treatment decreases CD115^+^ LY6C^hi^ cell population in bone marrow of tumor-bearing mice. C57/BL6 mice were inoculated with B16/F10 tumor cells. After 14 days, ATL-1 (1 μg/animal) or vehicle (20 μL ethanol) was administered. On 21st day, the animals were sacrificed, the femurs and tibias dissected, and the bone marrow cells isolated for further analysis by flow cytometry. **(A)** Representative dot plot used to quantify the percentage of CD115^+^/LY6C^hi^ and CD115^+^/LY6C^low^ populations within the selected gate. **(B)** CD115 ^+^ LY6C^hi^ (*n* = 7); **(C)** CD115 ^+^ LY6C^low^ (*n* = 7). Data are expressed as mean ± SEM. *p < 0.05; **p < 0.01; ***p < 0.001; ****p < 0.0001 by unpaired *t*-test or ANOVA test, followed by *Bonferroni* test.

We analyzed the effect of lipoxin treatment on the profile of mononuclear cells derived from the bone marrow of tumor-bearing mice. Bone marrow-derived monocytes were obtained 21 days after B16/F10 cells inoculation in the mice. We observed that ATL-1 injection on the 14th day after tumor induction decreased CD115^+^Ly6C^hi^ cells (Figures 2A,B) and increases CD115^+^LY6C^low^ populations in bone marrow (Figures 2A,C).

**Figure 3:**
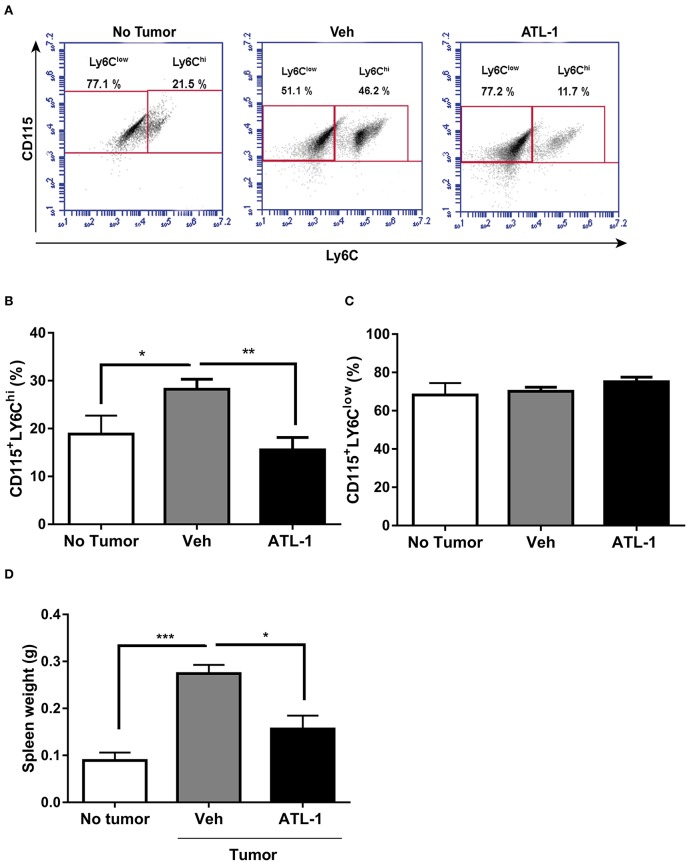
ATL-1 treatment decreases CD115^+^LY6C^hi^ monocyte population in the spleen of tumor-bearing mice. C57/BL6 mice were inoculated with B16/F10 tumor cells. After 14 days, ATL-1 (1 μg/animal) or vehicle (20 μL ethanol) was administered. On 21st day, the animals were sacrificed, the spleens dissected and weighted, and monocytes isolated for further analysis by flow cytometry. **(A)** Representative dot plot used to quantify the percentage of CD115^+^/LY6C^hi^ and CD115^+^/LY6C^low^ populations within the selected gate. **(B)** CD115^+^ LY6C^hi^ (*n* = 8); **(C)** CD115^+^ LY6C^low.^ (*n* = 8); **(D)** spleen weight (*n* = 6). Data are expressed as mean ± SEM. *p < 0.05; **p < 0.01; ***p < 0.001 by unpaired *t*-test or ANOVA test, followed by *Bonferroni* test.

During tumor development, myeloid cells can accumulate in the spleen (17, 27) making this organ an important source of tumor-infiltrating immune cells. As shown in Figures 3A,B, ATL-1 treatment also is able to decreases CD115^+^LY6C^hi^ monocytes population in the spleen from tumor-bearing mice, when compared to control animals. The effect of ATL-1 on the spleen, different from what occurred to bone marrow, was selective for those TAMs precursors, since ATL-1 did not alter the CD115^+^LY6C^low^ monocyte population (Figures 3A,C). Importantly, the reduction of CD115^+^LY6C^hi^ monocytes population was accompanied by a significantly decrease of the spleen weight in ATL-1-treated animals, that present values similar to non-tumor state (Figure 3D).

**Figure 4:**
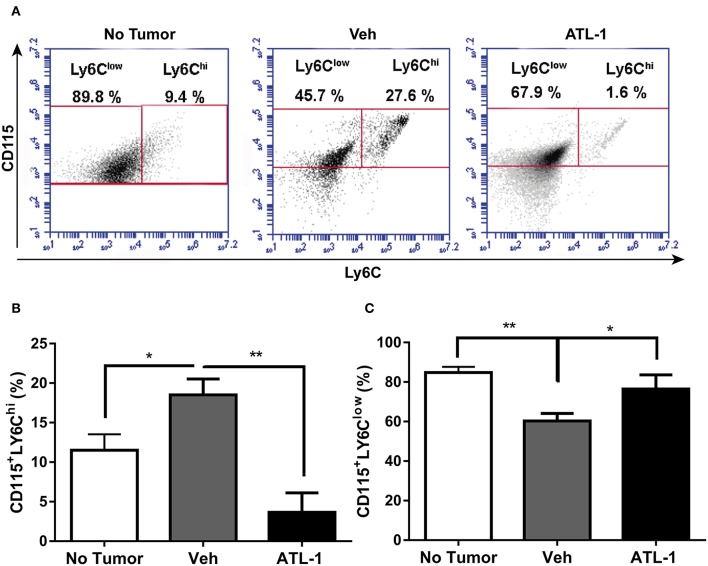
ATL-1 treatment decreases blood circulating CD115^+^LY6C^hi^ monocyte population of tumor-bearing mice. C57/BL6 mice were inoculated with B16/F10 tumor cells. After 14 days, ATL-1 (1 μg/animal) or vehicle (20 μL ethanol) was administered. On 21st day, the animals were anesthetized, and blood was withdrawn by cardiac puncture. Thereafter, monocytes were isolated from the blood for further labeling with specific antibodies for flow cytometry analysis. **(A)** Representative dot plot used to quantify the percentage of CD115^+^/LY6C^hi^ and CD115^+^/LY6C^low^ populations within the selected gate. **(B)** CD115^+^ LY6C^hi^ (*n* = 5–6); **(C)** CD115^+^ LY6C^low^ (*n* = 5–6). Data are expressed as mean ± SEM. *p < 0.05; **p < 0.01 by unpaired *t*-test or ANOVA test, followed by *Bonferroni* test.

Besides the bone marrow and spleen, the monocyte population from circulation was also analyzed. In Figures 4A,B, we observed that ATL-1 treatment significantly decreases blood circulating CD115^+^LY6C^hi^ monocyte population in tumor-bearing animals, compared with cells isolated from non-treated animals. Remarkably, ATL-1 treatment increased circulating CD115^+^LY6C^low^ monocytes (Figures 4A,C).

### ATL-1 Has a Specific Effect on Monocytes From Tumor-Bearing Mice

**Figure 5:**
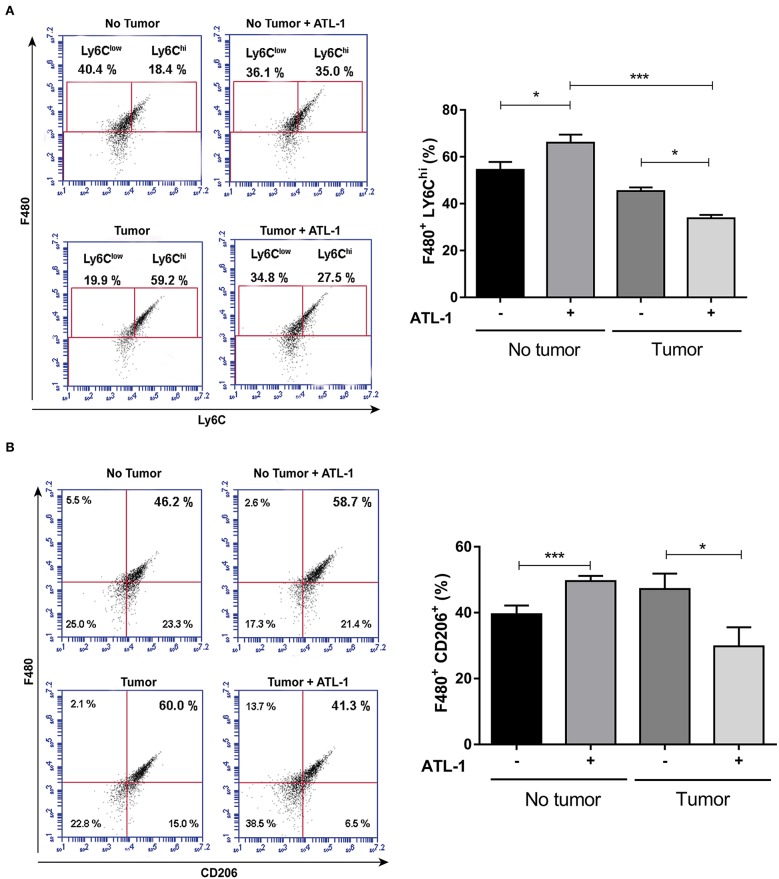
ATL-1 has a selective effect on monocytes from tumor-bearing mice. C57/BL6 mice were inoculated or not with B16/F10 tumor cells. After 14 days, animals with or without tumor, were anesthetized and blood was withdrawn by cardiac puncture. Monocytes were isolated from the blood, seeded in 96-wells plate, treated or not with ATL-1 (10 nM) for 3 days and analyzed by flow cytometry. **(A)** F480/LY6C^hi^ population 3 days after ATL-1 treatment (*n* = 6); **(B)** F480/CD206 population 3 days after ATL-1 treatment (*n* = 6). Data are expressed as mean ± SEM. *p < 0.05; ***p < 0.001 by ANOVA test, followed by *Bonferroni* test.

At 14th day after B16/F10 cells injection, blood monocytes were isolated by cardiac puncture, seeded on 96 wells plate and treated or not with ATL-1 (10 nM) for 3 days *in vitro*. After this time, adhered cells began to present macrophage markers (as CD206), but still maintaining blood monocyte markers (as LY6C). We observed that ATL-1 showed a specific effect, decreasing the expression of LY6C^hi^ (Figure 5A) and CD206 (Figure 5B) on monocyte/macrophage population derived from tumor-bearing mice blood, while increasing these markers on blood monocyte/macrophage from animals without tumors.

### ATL-1 Treatment Decreases M2 Markers and Increases M1 Markers Expressions of TAMs *in vivo*

**Figure 6:**
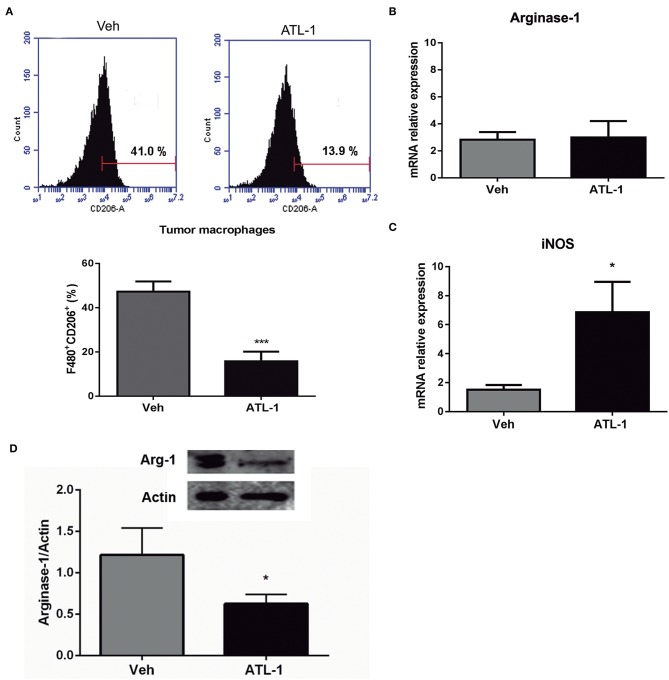
ATL-1 treatment decreases M2 markers and increases M1 markers expression of TAMs *in vivo***. ***C*57/BL6 mice were inoculated with B16/F10 tumor cells. After 14 days, ATL-1 (1 μg/animal) or vehicle (20 μL ethanol) was administered i.v. After additional 7 days, animals were sacrificed, the tumors were dissected, and TAMs isolated for further analysis by flow cytometry, western blotting and RTqPCR. **(A)** F480^+^/ CD206^+^ cells (*n* = 9); **(B)** Arginase-1 gene expression (*n* = 6); **(C)** iNOS gene expression (*n* = 6) and **(D)** arginase-1 protein expression (*n* = 4). Gene expression is relative to the expression on 14th day. GAPDH or actin were used as housekeeping genes. Data are expressed as mean ± SEM. *p < 0.05; ***p < 0.001 by unpaired *t*-test.

B16/F10 cells were inoculated (s.c) into C57/Bl6 mice and, after 14 days, animals received a single i.v. injection of ATL-1 (1 μg/mice). TAMs were isolated on day 21 from animals treated with ATL-1 or ethanol (vehicle) and the expression of M2 and M1 markers was investigated by flow cytometry, immunoblotting, and RTqPCR. TAMs isolated from tumors of mice treated with ATL-1 showed a decrease in classical M2 markers, as CD206 (Figure 6A) and Arginase-1 (Figure 6D). Noteworthy, despite there was no difference in arginase-1 gene expression (Figure 6B), the protein expression was significantly decreased in TAMs isolated from ATL-1-treated animals (Figure 6D). On the other hand, treatment with ATL-1 led to an increase on gene expression of iNOS (Figure 6C), a classical M1 marker.

### ATL-1 Treatment Diminished Tumor-Macrophage Infiltration and Is Able to Improve the Survival of Tumor-Bearing Mice

**Figure 7:**
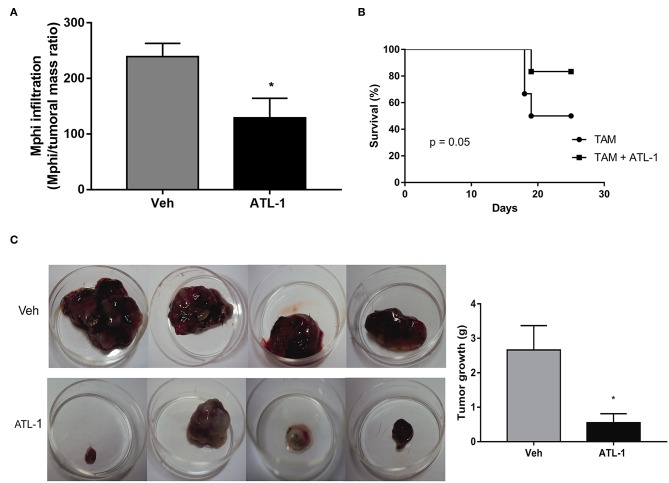
ATL-1 treatment diminished tumor-macrophage infiltration and is able to improve the survival of tumor-bearing mice. C57/BL6 mice were inoculated with B16/F10 tumor cells. After 14 days, ATL-1 (1 μg/animal) or vehicle (20 μL ethanol) was administered. On 21st day, some animals were euthanized, and the tumor extracted. Some animals were left in the cage until day 25 for the survival analysis. Mphi (macrophages) were isolated and counted using a Neubauer chamber. **(A)** The graph represents the number of tumor-infiltrating macrophages (*n* = 4–5). Data are expressed as mean ± SEM. *p < 0.05 by unpaired *t*-test; **(B)** Survival curve (*n* = 6), the survival was recorded according to Kaplan–Meier analysis and analyzed by log-rank (Mantel–Cox) test; **(C)** Representative pictures and quantitative analysis (weight) of tumors developed in mice treated (lower row) and non-treated (upper row) with ATL-1. Data are expressed as mean ± SEM. *p < 0.05 by unpaired *t*-test.

Since lipoxin analog altered the profile of monocyte population derived from bone marrow, it would be possible that ATL-1 would interfere with the ability of these cells to migrate toward the tumor. Therefore, we decided to evaluate the effect of ATL-1 macrophage population infiltrated into tumors in treated and non-treated animals, on the 21st day after melanoma cells inoculation. Figure 7A shows that ATL-1 induced a decrease in the macrophage number infiltrated in tumors.

Besides that, ATL-1 treatment increased the survival rate of tumor-bearing mice that reached more than 80% on the 23rd day after tumor injection (Figure 7B) and considerably impair the tumor growth (Figure 7C).

## Discussion

The main results of the present work, are (i) ATL-1 has systemic effects *in vivo*, selectively acting on LY6C^hi^ monocytes, precursors of TAMs; (ii) this cell population is decreased in the bone marrow, in the spleen and in the blood of tumor-bearing mice treated with ATL-1; (iii) ATL-1 treatment modified the phenotype of macrophages present in tumors, down modulating the M2-like cells toward a cytotoxic M1-like profile and iv) one single dose of ATL-1 substantially increased survival rate of tumor-bearing mice. It is well-established that macrophages within the tumor microenvironment contribute to an inflammatory milieu and favor cancer progression (1, 2, 28–30). Targeting these cells for therapeutic purposes has been extensively studied and positive results have been found in different experimental approaches. TAMs ablation or re-polarization proved to be beneficial in cancer therapy (26, 31, 32). This relationship between inflammatory cells and tumor development suggests that mediators with pro-resolving properties may be promising tools for controlling tumor progression. Lipoxins, as well as other pro-resolving mediators, such as the resolvins and maresins, are potential targets for cancer treatment. Our group has previously demonstrated *in vitro* that lipoxins have anti-inflammatory and pro-resolving effects on monocytes and endothelial cells (24, 25, 33). In monocytes, lipoxins exhibit specific stimulatory activities, promoting migration and adhesion and inhibiting cell apoptosis (24, 25, 34). In contrast, in endothelial cells, lipoxins inhibited proliferation and migration, inhibiting the angiogenic process (33). More recently, we reported that ATL-1, a stable analogous of 15-epi-lipoxinA4, selectively switch TAM phenotype *in vitro*, contributing to the impairment in tumor growth (26).

Recent studies have shown that LY6C^hi^ monocytes accumulate in tumors and renew part of TAM population in the tumor microenvironment (20). In addition, in cancer, extramedullary splenic hematopoiesis is also important to maintain the continuous supply of monocytes to the tumor (20, 27). Thus, therapies capable of altering the profile of these LY6C^hi^ monocytes before they reach the tumor microenvironment and become TAM, could inhibit tumor progression. Since the intravenous treatment allows lipoxin to act systemically, we evaluated the population of those potential TAM precursors, LY6C^hi^ monocytes, in blood, spleen, and bone marrow of tumor bearing mice. Corroborating data from the literature, animals with well-established tumors presented an increase in circulating LY6C^hi^ monocytes. ATL-1 treatment selectively decreased LY6C^hi^ monocytes population in the bone marrow, in the spleen, and in the blood of tumor-bearing mice. Curiously, the population of LY6C^low^ monocytes was significantly augmented only in blood and bone marrow of lipoxin-treated animals. This differential outcome may be probably due that ATL-1 reaches higher concentrations on circulation, before its distribution in tissues. Another possibility to the increased blood and bone marrow LY6C^low^ population is that this population in the spleen of mice treated with ATL-1 may be rapidly redistributed to the blood.

Ly6C^hi^ monocytes are classified as “classical” or “inflammatory” that present high expression of CCR2, contributing to their higher migratory capacity (35). These cells are large producers of proinflammatory cytokines, including TNF-α, and are more likely to mature as inflammatory M1 macrophages, able to kill neoplastic cells (19, 36). However, the tumor microenvironment can modify this profile, shifting cells toward a M2-like profile with pro-tumor characteristics. Alternatively, Ly6C^low^ monocytes are described as “non-classical” with low expression of CCR2, what decreases their ability to migrate toward the inflammatory focus. Ly6C^low^ cells have a great capacity of tissue repair, helping in the resolution of inflammation and in the return to tissue homeostasis (35–37). Several mechanisms are associated with TAM recruitment, differentiation and accumulation in tumors. Classically, it is dependent on bloodstream-derived monocyte infiltration mediated by CCL2, CCL7, CXCL12, VEGF, and other cytokines produced in the tumor microenvironment by stromal and tumor cells (19, 38). Other studies have reported the contribution of *in situ* TAM proliferation (20, 37). The spleen can also act as an extramedullar reservoir of monocytes, being an important source of Ly6C^hi^ cells that migrate toward the tumor and differentiate in TAM (21). We demonstrate herein that the treatment with ATL-1 decreased LY6C^hi^ and increased LY6C^low^ populations in blood, thus reducing the population of monocytes with the right profile to migrate toward the tumor. Because of that, it was expected that the ATL-1 treatment would diminish monocyte recruitment to the tumor, leading to a reduced TAM cellularity in this microenvironment. Furthermore, we have early demonstrated, using the same *in vivo* experimental model, that ATL-1 reduced the tumor size at the day 21, keeping the remaining tumor mass with equivalent size founded before lipoxin treatment (day 14) (26). Corroborating this data, we observed that the treatment with ATL-1 decreases the macrophage population in the tumor and impair tumor growth (Figures 7A,C).

In addition to the effect of ATL-1, by decreasing the monocytes that would migrate to the tumor and become TAM, we observed that ATL-1 treatment decreased the weight of spleens of tumor-bearing mice. This effect, as a possible consequence of a diminished spleen cellularity, may lead to lower TAM replacement in the tumor. In face of these results, we suggest that ATL-1, together with its ability in inhibiting the angiogenic process (33), also decreases the availability of TAMs precursor monocytes, able to migrate to the tumor. Additionally, ATL-1 is also capable of acting directly on TAMs, altering the M2-like profile of the remaining macrophages in the tumor toward an M1-like cytotoxic profile. Thus, ATL-1 actions lead to an impairment of tumor progression.

M1 macrophages are potent producers of cytotoxic molecules with important functions in defense against aggressive agents, including neoplastic cells (39). In these macrophages, iNOS is the main metabolic route of L-arginine, leading to the production of L-citrulline and NO, which responds in large part to the anti-tumor activity of macrophages (40). In the tumor microenvironment, TAMs preferentially express arginase-1, which competes with iNOS for L-arginine to form L-ornithine and urea. In turn, L-ornithine can be subsequently catalyzed by ornithine decarboxylase to form polyamines (such as putrescine, spermidine and spermine) (41), important to increase the ability of cancer cells to invade and metastasize while diminishing the antitumor immune functions of immune cells. High levels of arginase-1 can promote tumor growth through the production of L-ornithine and putrescine. Furthermore, the reduction in NO production decreases macrophage cytotoxicity, and suppress T-cell antitumor activity (42–44).

ATL-1 treatment enhanced *in vivo* iNOS gene expression in TAMs, as it did in an *in vitro* setting (26). Although the gene expression of arginase-1 in these cells was not altered, we observed a lower protein translation of this enzyme in ATL-1 treated animals. In contrast, the gene expression of iNOS in TAMs from animals treated with ATL-1 is almost 4-fold greater than arginase-1 gene expression, resulting in an increase in the iNOS/arginase-1 ratio. In this scenario, iNOS is an advantage in the competition for the substrate, increasing NO production by TAMs, and preventing the production of L-ornithine and polyamines by arginase-1, besides being cytotoxic for tumor cells.

TAMs usually present an upregulation of cell-surface scavenger receptors, such as the mannose receptor (MRC1/CD206), which contribute to tumor immunosuppression, angiogenesis and metastasis (45). ATL-1 treatment decreased CD206^+^ TAMs in tumor-bearing mice, contributing for the shift of M2-like TAMs toward an antitumoral profile *in vivo*.

Together our results point out an important effect of lipoxins in several stages of the inflammatory response to the tumor, regulating the monocyte/macrophage activation profile *in situ*, as well as in sites far from the tumor microenvironment. Therefore, our study suggests that lipoxins, as well as other anti-inflammatory and pro-resolving lipid mediators, may have a substantial role in the tumor control, allowing the development of new tools in the cancer treatment.

## Data Availability

All datasets generated for this study are included in the manuscript and/or the Supplementary Files.

## Ethics Statement

This study was carried out in accordance with the recommendations of Committee for the Ethics of Animal Experimentation of the Universidade do Estado do Rio de Janeiro (UERJ, Permit number: CEA/074/2012). The protocol was approved by this committee.

## Author Contributions

Nd-B, RS, and CB-F conceptualized the study. Nd-B performed the experiments, analyzed the data, and drafted the manuscript. Hd-C contributed to data acquisition. RS and CB-F supervised the study and revised the manuscript and also had full access to all data, taken the responsibility for the integrity and the accuracy of the data analysis. CB-F is the guarantor of this work. All authors read and approved the final manuscript.

### Conflict of Interest Statement

The authors declare that the research was conducted in the absence of any commercial or financial relationships that could be construed as a potential conflict of interest.

## Abbreviations

APC: Allophycocyanin
ATCC: American Type Culture Collection
ATL-1: stable analog of aspirin-triggered lipoxin – 15-epi-LXA_4_
cDNA: complementary DNA
ECM: Extracellular matrix
FACS: Fluorescence-activated cell sorting
FITC: Fluorescein Isothiocyanate
Hi: high
iNOS: Inducible nitric oxide synthase
LX: Lipoxin
NO: Nitric oxide
NOS2: Nitric oxide synthase 2
PCR: Polymerase Chain Reaction
PE: Phycoerythrin
ROS: Reactive oxygen species
TAM: Tumor-associated macrophage
TNF-α: Tumor Necrosis Factor Alpha
VEGF: Vascular endothelial growth factor.
