# Supplementary figures and images for: Lipoxin-Induced Phenotypic Changes in CD115+LY6Chi Monocytes TAM Precursors Inhibits Tumor Development
Source: Front Oncol. 2019 Jun 19;9:540. doi: 10.3389/fonc.2019.00540 (PMC6593314; doi:10.3389/fonc.2019.00540)

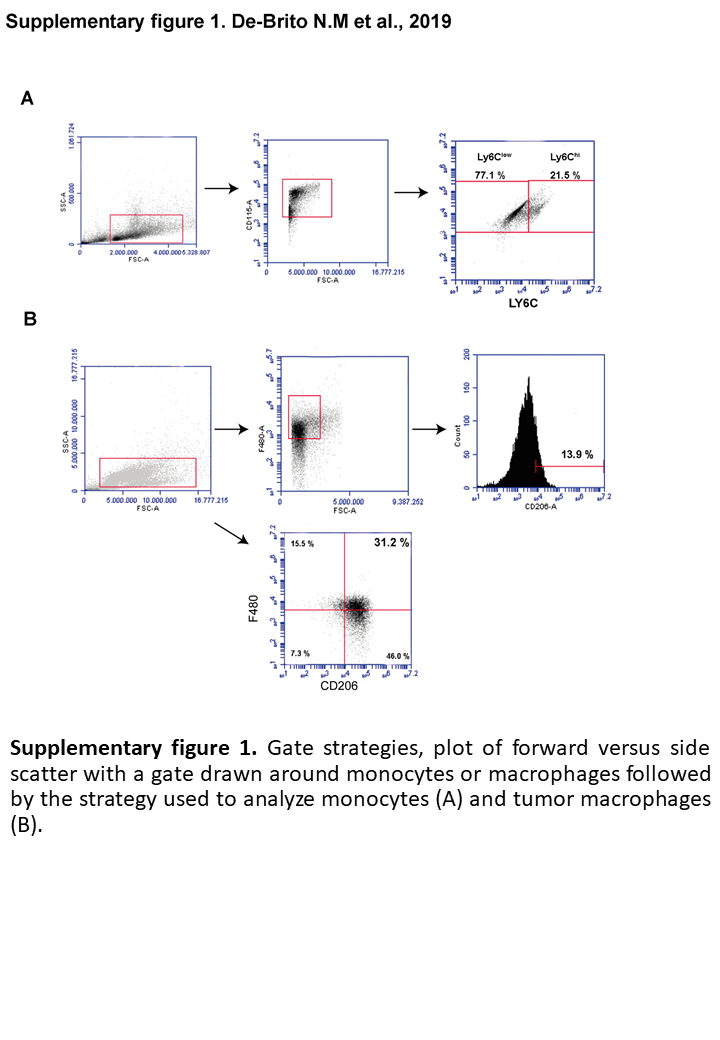

Supplement: Supplementary file 1 [file Image_1.TIF]
